# Development of a simulation and skills centre in East Africa: a Rwandan-Canadian partnership

**DOI:** 10.11604/pamj.2014.17.315.4211

**Published:** 2014-04-25

**Authors:** Patricia Livingston, Jonathan Bailey, Georges Ntakiyiruta, Christian Mukwesi, Sara Whynot, Peter Brindley

**Affiliations:** 1 Dalhousie University, Department of Anesthesia, Pain Management and Perioperative Medicine, Halifax, Nova Scotia, Canada; 2 Dalhousie University, Department of Surgery, Halifax, Nova Scotia, Canada; 3 University of Rwanda, Department of Surgery, Kigali, Rwanda; 4 University of Rwanda, Department of Anesthesia and Intensive Care, Kigali, Rwanda; 5 University of Alberta, Critical Care Medicine, Edmonton, Alberta, Canada

**Keywords:** Simulation, Medical Education, Rwanda, Low-Income Country

## Abstract

Simulation replicates clinical experiences without patient risk; it remains uncommon in lower-income countries. We outline the creation of Rwanda's first centre for simulation and skills training. We secured funding for renovations, equipment and staff; curricula were developed, tested, and refined; local clinicians were trained to teach. In 13 months the centre provided 2,377 learning-encounters and 822 hours of training to Rwandan health care professionals. Our strategy represents an adaptable model for simulation and skills centre development in low-resources settings

## Brief

I hear and I forget; I see and I remember; I do and I understand. Confucius (500 BC) Simulation replicates clinical experiences in an interactive, immersive and reflective manner: ideally suited for adult learners and without risk to patients [1]. Simulation for medical education in well-resourced settings has grown exponentially, [2] but remains uncommon in lower-income countries. Rwanda is a small central African country where nearly one million people died during the 1994 genocide. Health professionals were not spared and only one physician anaesthetist remained for years after [3]. Despite impressive recovery, a 2010 survey found a population of 10.1 million had only 45 full time surgeons and 12 physician anaesthetists (or 0.12 physician anaesthetists and 0.45 surgeons per 100,000 populations) [4]. In comparison, Canada has 35 million people, 9,148 surgeons, and 3,023 physician anaesthetists (or 9 physician anaesthetists and 26 surgeons per 100,000 populations) [5]. In order to aid Rwanda's re-growth, our multidisciplinary partnership of Canadian and Rwandan health professionals created the country's first medical simulation and skills training centre. This manuscript provides a template for others with similar intent.

Rwandan medical and nursing training previously relied upon practice on real patients, minimal direct supervision, and passive classroom learning [3, 4]. This traditional way of training has predominated worldwide. However, healthcare is increasingly understood as a complex social system, where human factors (such as communication and teamwork), and other non-technical skills, are the greatest determinants of safety [6]. These skills are difficult to teach in the operating theatre or in a classroom, but comparatively easy with simulators. [7] Recognizing the benefits of simulation-based education, the shortage of clinical teachers, and the importance of non-technical skills, we established a centre for simulation in Rwanda.

**Figure 1:**
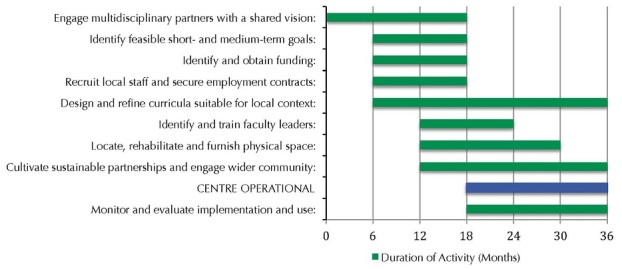
Activities and timelines for successful implementation of the Faculty of Medicine Simulation and Skills Centre at the University Teaching Hospital of Kigali

Figure 1 outlines key activities and project timeline in developing Rwanda's Faculty of Medicine Simulation and Skills Centre. The long-standing relationship (since 2006) between the University of Rwanda and the Canadian Anesthesiologists’ Society International Education Foundation [8] provided a strong foundation. Rwandan partners brought knowledge of their capacity and needs; Canadian partners accessed resources such as teachers, funding, equipment, and curricula. The team used an iterative process to develop mission and vision statements, to outline the rationale for simulation in Rwanda, to identify learners, and to create contextually relevant courses. This systematic approach helped secure a $100,000 grant from Grand Challenges Canada.

Next, we turned to practical details: space, staff, equipment, and curriculum. A two-room building (with proximity to teachers and learners) was secured at the University Teaching Hospital of Kigali. Infrastructure upgrades were completed on the lighting, plumbing, and cabinets. The centre now allows for flexible setup, i.e. operating room, emergency bay, or obstetrical suite. A full-time program coordinator, part-time medical director, and an assistant medical director were hired. The medical director and assistant medical director received simulation training at the Scottish Clinical Simulation Centre. A Canadian simulation program coordinator maintains regular contact in order to mentor her Rwandan counterpart.

Simulation is better understood as a technique (a way of teaching), rather than just a technology (a piece of equipment). Accordingly, skills acquisition is not significantly increased with more expensive simulators. Moreover, psychological fidelity (the degree to which the task feels “real”) may be more important than physical fidelity (the specific device used) [1]. We also capitalized on local experience by teaching surgical-skills with inexpensive locally sourced materials [9]. We secured low-fidelity simulators including intubation heads, resuscitation dolls, and simple task-trainers. Our team designed templates for teaching clinical skills (e.g., intubation, creating a stoma) and clinical scenarios for team training (e.g., massive trauma, ruptured uterus). The scenarios included pre-determined goals from the domains of medical knowledge, manual skills and non-technical skills. Rwandan partners ensured the curriculum was locally relevant. We conducted a train-the-trainer course to prepare educators for teaching both the technical and non-technical aspects of each scenario. Educators consolidated their teaching skills (and faculty refined the scenarios) by presenting the same material to students. This was followed by structured-feedback to students and further dialogue between faculty and learners.

Table 1 outlines the first 13 months of utilization data that represents 2377 learning encounters and 822 hours of instructional time. Multidisciplinary users include anaesthesia, surgery, emergency medicine, obstetrics and gynecology, pediatrics, and nursing. Participant feedback indicates a high level of satisfaction, and that the cultivation of long-term relationships, and the sense of partnership, was key.

**Table 1:** Utilization data, by specialty, from February 1, 2013 – February 28, 2014

Learning Opportunities		2377
Hours of Use		822
**Use by Specialty**	Anesthesia	727
	Surgery	768
	Obstetrics/Gynecology	177
	Emergency Medicine/ General Practice	214
	Pediatrics	78
	Ear, Nose and Throat	1
	Nursing	189
	Other	195

It remains to be seen if these successes will be maintained long-term. We believe our experience offers face-validity to the assertion that simulation can be applied in low-income jurisdictions by offering an inexpensive, flexible and practical form of experiential learning that can be tailored to suit local needs.
